# Comparing *Nigella sativa* Oil and Fish Oil in Treatment of Vitiligo

**DOI:** 10.5812/ircmj.4515

**Published:** 2014-06-05

**Authors:** Alireza Ghorbanibirgani, Ali Khalili, Darioush Rokhafrooz

**Affiliations:** 1 Faculty Member of Nursing, Department of Nursing and Midwifery, Gachsaran Branch, Islamic Azad University, Gachsaran, IR Iran; 2 Shahid Beheshti University of Medical Sciences, Tehran, IR Iran

**Keywords:** Nigella sativa, Fish Oils, Vitiligo

## Abstract

**Background:**

Vitiligo is one of the autoimmune skin diseases that destroy the melanocytes of the skin. Moreover, its prevalence varies in different countries and regions.

**Objectives:**

The aim of this study was to compare the effect of *Nigella sativa* and fish oil on vitiligo lesions of the patients referred to a dermatology clinic.

**Materials and Methods:**

This randomized, double blind clinical trial was conducted in the dermatology clinic of the Imam Khomeini Hospital Ahvaz, Iran, from June to December 2011. We used a randomized simple sampling. From 96 patients with vitiligo, 52 eligible patients were selected and allocated to two groups with equal size. The study medications were applied twice a day by patients on their lesions. After six months, the improvement rate of lesions was assessed by the Vitiligo Area Scoring Index (VASI). Data were analyzed using SPSS v. 15; P value < 0.05 was considered as statistically significant.

**Results:**

After six months, a mean score of VASI decreased from 4.98 to 3.75 in patients applying topical *Nigella sativa* and from 4.98 to 4.62 in those using topical fish oil. Most of the percent improvement observed in upper extremities, trunk, head, and neck of those who received *Nigella sativa* and head, neck, trunk, and feet of those who received fish oil. No adverse effect was reported by the patients.

**Conclusions:**

*Nigella sativa* oil and fish oil were effective in reduction the size of patient’s lesions; however, *Nigella sativa* was more effective in comparison to the fish oil. Therefore, using *Nigella sativa* with the major drugs in the treatment of vitiligo is recommended.

## 1. Background

Vitiligo is a skin disease that causes white spots due to loss of skin pigment cells. In vitiligo, melanocytes (cells that produce pigment) of the skin, mucous membrane, and the retina are damaged that causes white spots in different areas of skin (1). This disease is the most common disorder of the skin pigmentation with an incidence rate of 0.1% to 2% in different populations (2-4). The symptoms of the disorder manifest before the age of 20 in about half of the patients. Approximately, one-fifth of the family members of the patients are affected by vitiligo. The disease affect all races and genders equally (1) and is more common in people with other autoimmune disorders (3). This disease is more likely to develop in late childhood (9-12 years old) through middle age (1). Vitiligo lesions usually create milky and white spots that are usually seen symmetrically and bilaterally. The most commonly affected areas of the skin include the face, lips, hands, arms, feet, and the genitals. Moreover, the color of the hairs that grow in the affected areas is usually white (2). Although the main cause of this disorder is not known yet, possible mechanisms such as genetic, autoimmune, biochemical, nervous, and viral factors are proposed as probable causes (1-3). Many studies concerning vitiligo and its treatment have been conducted; however, no unique effective treatment that may improve all patients has been identified yet. Topical treatments are preferred in patients with vitiligo. The use of herbal medications is among the effective treatments that has had useful effects in treating vitiligo lesion. In this context, different studies have been conducted on various herbal medications worldwide to control as well as to treat vitiligo. Among the herbal medications, *Ginkgo biloba* (5, 6), *Nigella sativa* (Black cumin) (7-9), and black pepper (10) can be noted. They have been used in several studies and had different effects on patients with vitiligo. Caraway plant (*Carum carvi*) is widely used to treat various diseases in the Asian and European countries. Thymoquinone is a major component of the seeds of this plant, i.e. *Nigella sativa*, that has anti-inflammatory, antimicrobial, antioxidant, and antitumor effects as well as the strengthening effects on the immune system; in fact, the presence of α-linolenic acid (ALA) and stearidonic acid (SDA) enhances the immune response, especially in T cells (11-19). This medication is used to treat kidney disease (20, 21), high blood pressure (22-24), rheumatoid arthritis (25), diabetes mellitus (26-28), allergic rhinitis (29), asthma (30, 31), and osteoporosis (32). Fish oil is also a rich source of omega-3 fatty acids including eicosapentaenoic acid (EPA) and docosahexaenoic acid (DHA), which are among the essential fatty acids. In the human body, EPA and DHA are converted to a series of active chemical compounds called eicosanoids that have anti-inflammatory properties. Another advantage of fish oil is related to its vitamin E component. Vitamin E is a potent antioxidant that helps to protect cells against free radical damage. It also helps to process of fatty acids and to improve the health of the hair and skin (33-38); moreover, it is used in the treatment of various diseases (39-41).

## 2. Objectives

This randomized, double blind, controlled clinical trial was conducted to compare the effect of *Nigella sativa* oil with fish oil on improvement of the vitiligo lesions.

## 3. Materials and Methods

Patients with vitiligo referred to the dermatology clinic of Imam Khomeini Hospital, Ahvaz, Iran, from June to December 2011 were assessed. Randomization was performed by using a simple random table. A total of 96 patients were examined by a dermatologist. We included patients with generalized or localized vitiligo for less than five years. The patients’ disease was confirmed by a dermatologist. Exclusion criteria included positive family history of vitiligo, pregnancy (in women), and allergy to *Nigella sativa* and/or fish oil. Finally, 52 patients were recruited after signing a written informed consent form. They were randomly allocated to two 26-patient groups. *Nigella sativa* seeds were purchased from the local herbalist in Ahvaz. The seeds were authenticated by Pharmacognosy Department, School of Pharmacy, Isfahan University of Medical Sciences, Iran. Seeds were cleaned, dried, mechanically smashed and powdered, extracted with 96% ethanol, and evaporated with rotary evaporator to render the extract alcohol-free. The extracts were transferred into tubes, each containing 100 g of *Nigella sativa*. The above procedures were undertaken in the Giah Essence Phytopharm Co, Gorgan, Iran. Thus, *Nigella sativa *oil and fish oil were prepared in quite similar containers (100 g container) by a pharmacologist at pharmacy. The A (*Nigella sativa* oil) group applied *Nigella sativa* oil to the areas of skin lesions and the B (Fish oil) group received fish oil twice a day for six months. Seven visit sessions were made for the patients entering the study by one observer: one before the study and the rest at the end of each month through the sixth month of treatment. During the study, the participants and the researcher were unaware of the drug in tubes. A form was prepared for each patient in which the data were recorded. During the treatment period, skin lesions of patients were evaluated by observers using Vitiligo Area Scoring Index (VASI) tool. The percentage of vitiligo involvement was calculated in terms of hand units. One hand unit, which encompasses the palm plus the volar surface of all digits, is approximately equivalent to 1% of the total body surface area. The degree of pigmentation was estimated at the nearest of the one of the following: 100%, complete depigmentation, i.e. no pigment is present (score = 5); 75%, depigmented area exceeded the pigmented area (score = 4); 50%, pigmented and depigmented areas were equal (score = 3); 25%, pigmented area exceeded depigmented area (score = 2); 10%, only specks of depigmentation were present (score = 1); and no depigmentation (score = 0). Patients were assessed for percentage of pigmentation at the time of entering to the study, as well as at the end of each month during study by VASI (minimum score of 0 = no depigmentation; and maximum score of 5 = no pigmentation).

The validity and reliability of VASI have been reported previously (42-45). In order to gain reliability of VASI in this study, test-retest method with one month interval was used in which 15 patients from each group were asked to record the intensity, size, and quantity of depigmentation. The correlation coefficient was calculated to be 89%. Necessary warnings such as avoiding the use of the medications in the wounded areas, preventing drug exposure to the eyes, and washing the hands with water after application of the drug were given to the patients. The patients were also asked to inform the researcher if any side effects were noticed. To comply with the ethical issues, the study was conducted on the patients in coordination with physicians treating the patients and approval of the Ethics Committee of Gachsaran Azad University (ethical code B-52/324-011, date 14/04/2011). After getting permission from the Ethics Committee, the interested patients were invited to participate in this study by providing information for them about the medications and the goals of the study. Furthermore, patients were allowed to withdraw the study at any time they were not willing to continue working with the team. The data obtained from the study were used in order to assess the skin lesions intensity changes between two groups by using of descriptive statistical methods (percent¸ frequency, and mean ± standard deviation). Repeated measurement of ANOVA test was used in the cases of having normal distribution and otherwise, Friedman test was used. In this study, we used SPSS v.15 (SPSS Inc., Chicago, Illinois, USA) and P value < 0.05 was considered as statistically significant in all tests.

## 4. Results

After six months of treatment, all patients in both groups completed the study without attrition. Data from all the 52 patients, 19 (40%) female and 33 (60%) male, were analyzed. The mean age of participants was 43.65 ± 3.21 years, with 41 ± 4.62 years in men and 47 ± 2.18 years in women. The mean age at the onset of disease was 4.11 ± 1.46 years. Thirty nine patients (75%) were categorized as the generalized and 13 (25%) patients as localized type of vitiligo. After six months, the mean VASI score decreased from 4.98 ± 4.81 to 3.75 ± 3.91 in the *Nigella sativa* oil group (Table 1), and from 4.98 ± 4.80 to 4.62 ± 4.36 in the fish oil group (Table 2). The correlation coefficient (r) of Nigella sativa and fish oil effects in two study groups is presented in Table 3. The highest percentage of improvement in the Nigella sativa oil group was observed in the lower extremities, trunk, head, and neck; and in the fish oil group in the head and neck, trunk, and feet. The lowest percentage of improvement in the Nigella sativa oil group was observed in the head, neck, and hands; and in the fish oil group in the hands and lower extremities. No specific side effects were reported by patients during six months of treatment.

In a pilot study on 10 patients, sample size was calculated according to the following formula by two mean comparison (α= 0.05 and β = 0.2) as 52 and the patients were screened consecutively.

n = (Z _(1-β)_ + Z _(1-α/2)_)^2^ (δ_1_^2^ + δ_2_^2^)/(µ_1_ - µ_2_)^2^

**Figure 1.**
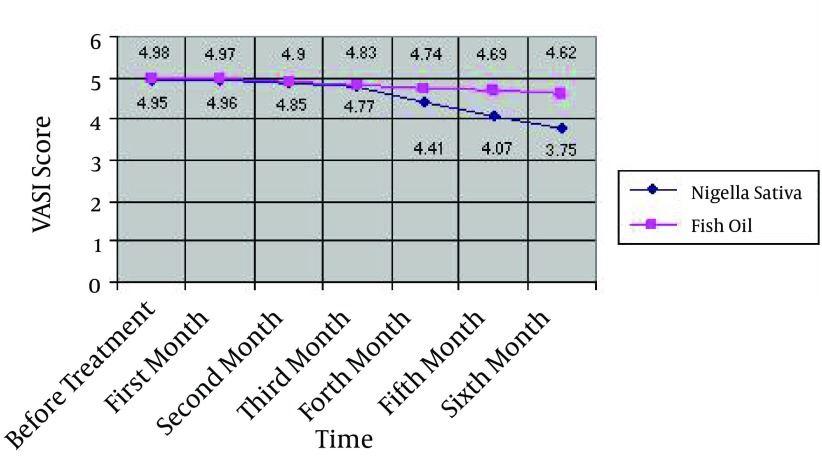
Reduction of VASI Score in *Nigella sativa* and Fish Oil Groups During the Six Months of Study

Repeated measures of ANOVA test indicated that there was no significant difference between reduction of VASI score in Nigella sativa oil and fish oil groups from the first through the third month of the study; however, there were significant differences in reducing VASI score in favor of Nigella sativa oil from the fourth through the sixth month (P < 0.020; Figure 1).

**Table 1.** Vitiligo Area Scoring Index Scores in Nigella sativa Group ^a,b^

Weeks of Treatment	*Nigella sativa* Oil	P Value
	VASI Score (Mean ± SD)	
**Before Treatment**	4.98 ± 4.81	0.094
**First Month**	4.96 ± 4.75	0.092
**Second Month**	4.85 ± 4.63	0.084
**Third Month**	4.77 ± 4.51	0.080
**Fourth Month**	4.41 ± 4.28	0.048
**Fifth Month**	4.07 ± 4.11	0.034
**Sixth Month**	3.75 ± 3.91	0.020

^a^ Abbreviation: VASI, vitiligo area scoring index.

^b^ There was no significant difference in reductions of VASI score between *Nigella sativa* and fish oil from the first through the third month of the study; however, there were significant differences in reduction of VASI score in favor of *Nigella sativa* after the fourth month (P < 0.020).

**Table 2.** Vitiligo Area Scoring Index Scores in Fish Oil Group^a,b^

Weeks of Treatment	Fish Oil	P Value
	VASI Score (Mean ± SD)	-
**Before Treatment**	4.98 ± 4.80	0.093
**First Month**	4.97 ± 4.78	0.091
**Second Month**	4.90 ± 4.72	0.079
**Third Month**	4.83 ± 4.59	0.081
**Fourth Month**	4.74 ± 4.54	0.078
**Fifth Month**	4.69 ± 4.43	0.075
**Sixth Month**	4.62 ± 4.36	0.067

^a^ Abbreviation: VASI, vitiligo area scoring index.

^b^ There was no significant difference between reduction of VASI score in fish oil group from the first through sixth month of the study (P < 0.067).

**Table 3.** Correlation Between *Nigella sativa* and Fish Oil With Improvement of Vitiligo Lesions

-	*Nigella sativa* Group	Fish Oil Group
**Correlation Coefficient (r)**	0.864	− 0.489
**P Value**	0.020	0.067

There was no significant difference between reductions of VASI score in *Nigella sativa* with fish oil groups during the first through the third month of the study; however, after the fourth month, there were significant differences in reduction of VASI score in favor of *Nigella sativa*.

## 5. Discussion

Vitiligo is an annoying and frustrating skin disease. It even affects the patients’ confidence and quality of life. Although various drugs are prescribed for these patients, no single drug that can reduce symptoms and skin lesions has been discovered. The most effective available drugs are herbal medications, which at least might have no side effect in comparison to chemical drugs and the patient can use them without fear of disturbing side effects.

This study was performed in order to compare the effect of *Nigella sativa* oil and fish oil on improving vitiligo lesions. Skin lesions of the patients were measured in determined times. After applying topical *Nigella sativa* oil and fish oil for six months, the total VASI score showed a significant improvement from baseline through the end of the study. The use of these drugs might reduce the size of lesions in patients and both drugs showed a downward trend in the VASI score from baseline to end of treatment (Figure 1). By searching databases such as PubMed, no similar study was found in this regard and our study seems to be the first study concerning treatment of vitiligo disease in humans by *Nigella sativa*. The latest study, which was done on animals in 2011 on the efficacy of *Nigella sativa* extract on animals, shows that applying drug on a lizard caused an increase in pigmentation and led to a darkening of the animal skin (8). In the present study, the depigmented areas were reduced over time and the skin color showed improvement. One reason for this positive response to treatment is the thymoquinone component of *Nigella sativa*. Thymoquinone, the main constituent of *Nigella sativa* seeds, protects cells against oxidative damage induced by a variety of free radical-generating pathologies (46). Thymoquinone can simulate the activity of acetylcholine, which causes the release of melanin and darkening of the skin through stimulation of cholinergic receptors (8). In addition, *Nigella sativa* oil administration was tolerable as well as safe and improved oxidative stress and clinical condition of patients (47). Fish oil could also make a good improvement by reducing the lesions size within six months although this recovery was less significant than that of *Nigella sativa*. In a study that was performed on 39 patients with chronic psoriasis in Birjand, the fish oil had the same effect in reducing skin lesions size in comparison to a combination of salicylic acid and betamethasone (40). The positive effect of fish oil on skin diseases such as vitiligo was indicated in another study in India (48). One reason that fish oil affects the skin diseases is the presence of omega-3 fatty acids that can prevent drying and scaling of skin in eczema, acne, and other skin diseases. In addition, it accelerates the recovery from skin diseases. Moreover, fish oil led to synthesis of anti-inflammatory properties in the skin as the result of EPA and DHA found in the phospholipid membrane. In the current study, administration of *Nigella sativa* and fish oil significantly decreased skin lesions size, indicating an improvement in clinical condition. A previous study has shown similar effects of *Nigella sativa* administration to an animal (8). The strong point of the present study is that it was performed in Iran for the first time and hypothesized that a combined method (*Nigella sativa* and fish oil) could be effective and desirable for improving and treating skin lesions. It was also shown that this type of treatment has no significant side effects and resulted in high patient satisfaction and acceptance. It is necessary to conduct long-time researches with larger sample size and different medication doses in order to confirm current findings. There were limitations to our study; for instance, this study was conducted in one clinic that might not be the representative of all the patients and hence, the results could not be generalized. Furthermore, some of the factors influencing skin lesions such as nutrition were uncontrollable. In summary, considering the distinct effect of fish oil and *Nigella sativa* oil on symptoms of patients with vitiligo, it is recommended to use herbal medications in treating these patients, especially since they have no significant side effects.
